# Mental well-being among the oldest old: revisiting the model of healthy ageing in a Finnish context

**DOI:** 10.1080/17482631.2020.1734276

**Published:** 2020-03-02

**Authors:** Johanna Nordmyr, Johanna Creswell-Smith, Valeria Donisi, Elvira Lara, Natalia Martín-María, Linda Nyholm, Anna. K. Forsman

**Affiliations:** a Faculty of Education and Welfare Studies, Health Sciences, Åbo Akademi University, Vaasa, Finland; b Mental Health Unit, Finnish Institute for Health and Welfare, Helsinki, Finland; c Section of Clinical Psychology, Department of Neurosciences, Biomedicine and Movement Sciences, University of Verona, Verona, Italy; d Department of Psychiatry, Universidad Autónoma de Madrid, Madrid, Spain; e Instituto de Salud Carlos III, Centro de Investigación Biomédica en Red de Salud Mental, CIBERSAM, Madrid, Spain; f Department of Psychiatry, Instituto de Investigación Sanitaria Princesa (IIS-princesa), Hospital Universitario de La Princesa, Madrid, Spain

**Keywords:** Older adults, oldest old, mental well-being, content analysis, focus groups, Scandinavia, Finland

## Abstract

**Purpose**: This study aimed to examine how participants aged 80 years old or over describe their mental well-being—exploring the suitability of the model of healthy ageing when outlining the mental well-being concept.

**Methods**: Six structured focus group interviews with 28 participants were conducted in Western Finland in 2017. Qualitative content analysis was performed, where both manifest and latent content was considered in a process involving meaning condensation and coding, followed by categorization.

**Results**: The healthy ageing model constituted a useful framework for the conceptualization of mental well-being, illustrating the links between these two constructs. The analysis resulted in a four-dimensional model of mental well-being in oldest old age, the key components being: *Activities*—enjoyment and fulfilment; *Capability*—functioning and independence; *Orientation*—awareness, shifted perspectives and values; and *Connectedness*—sense of belonging.

**Conclusions**: Although functional status plays an important role for well-being in general, it is not the principal component of self-reported mental well-being within the heterogeneous group of the oldest old. Further, many persons in this age group do not view themselves as passive or dependent, on the contrary, they underline the importance of empowering attitudes, a positive mindset and actively creating circumstances which support their mental well-being.

## Introduction

Research on ageing covers various aspects of the ageing process, with theoretical concepts such as healthy ageing (Guralnik & Kaplan, 1989) and successful ageing (Rowe & Kahn, 1997) guiding research endeavours in this growing field of interest. These two concepts include various definitions and models ranging from biomedical to biopsychosocial (Bowling & Dieppe, 2005; Peel, Bartlett, & McClure, 2004), with an overall emphasis on the maintenance of functioning. The previously dominating biomedical model focuses on physical processes and functioning, such as pathology and physical impairment, and does not consider the role of, for example, social participation and life satisfaction for health, nor the vast heterogeneity in health status in the population (Carver & Buchanan, 2016; Huppert, 2014; Stewart-Brown, 2013). Therefore, a healthy ageing model primarily based on a biomedical perspective or physical and functional ability will not fully explain many aspects of the ageing process, including the social and psychological aspects, where a more holistic and multi-dimensional approach is needed. In response, the World Health Organization launched a renewed definition of healthy ageing: *“the process of developing and maintaining the functional ability that enables well-being in older age”*. While functional ability is yet again in focus, the scope was broadened and reframed to cover “*having the capabilities that enable all people to be and do what they have reason to value”* (Beard et al., 2016; World Health Organization, 2015).

While contemporary concepts of healthy ageing may be expanding to encompass more positive, resource-focused perspectives, such as preserving and improving opportunities for social and mental well-being, quality of life, and life-course transitions, most empirical research on ageing still tends to focus on negative aspects such as mortality, morbidity, and disability (Cosco et al., 2014; Depp & Jeste, 2006). This is also reflected in a lack of mental health research focusing on older adults (e.g., Forsman, Ventus, van der Feltz-cornelis, & Wahlbeck, 2014), especially research with a focus on mental well-being in oldest old age (e.g., Miret et al., 2015).

Mental well-being has been conceptualized in various ways highlighting the multidimensionality of the concept. Diener (1984) has earlier depicted subjective well-being as consisting of cognitive (judgement about one’s life satisfaction) and affective (balance between positive and negative emotions) aspects. Seligman (2011) on the other hand introduced a model where psychological well-being encompassed the following domains: positive emotions, engagement, relationships, meaning, and accomplishment. Similarly, Ryff (2014) described the core dimensions of psychological well-being as autonomy, environmental mastery, purpose in life, personal growth, positive relationships with others and self-acceptance. Regarding older adults and mental well-being, a paradox has been highlighted in the literature—even though ageing is associated with increased risks of disability and ill-health, mental well-being tends to increase from middle age to very old age, even though this phenomenon may be somewhat domain-specific and dependent upon utilized measures (Hansen & Slagsvold, 2012; Kusumastuti et al., 2016; Steptoe, Deaton, & Stone, 2015). It is therefore fitting to explore the key aspects and causal mechanisms of mental well-being in later life, including a focus on the oldest old age group and their own experiences. This limited but growing body of research is needed in order to meet contemporary challenges related to the demographic transition and ageing society. Supporting and increasing mental well-being into oldest old age—including the psychosocial (Bowling, 2007) aspects of health alongside physical aspects (Beard et al., 2016)—is an important and timely line of enquiry. Including a stronger focus on mental well-being could further bolster the healthy ageing approach and offer unique opportunities for interventions and policy development (Cresswell-Smith et al., 2019).

The theoretical framework of healthy ageing is a growing area of interest also in Finland (e.g., Nosraty, Jylhä, Raittila, & Lumme-Sandt, 2015; Sarvimäki, 2015). This study endorses a positive view of healthy ageing as a multidimensional theoretical concept, involving a variety of physical, psychological, and social factors (Hansen-Kyle, 2005), and recognizes the influence of socio-cultural and societal factors, as well as physical, cognitive, psychological, social, and spiritual resources. Hence, mental well-being is in this study recognized as an integral part of healthy ageing, deserving more attention in the conceptualization. To the authors’ knowledge, there are no current research initiatives which aim to conceptualize mental well-being in oldest old age in the healthy ageing framework specifically in the Finnish context.

As a backdrop for this study, we consider the frequently cited publication by Bryant, Corbett, and Kutner (2001), where grounded theory-type methods were used to develop a model of healthy ageing covering four components: meaningful activities; balance between abilities and challenges; appropriate external resources; and personal attitudinal characteristics. This was an important contribution to the literature on healthy ageing, showcasing the multidimensional nature of the concept and including factors often seen as part of the mental well-being concept. The study highlights psychosocial perspectives of ageing and the process of adaptation into older stage of life in terms of “doing and having” as well as “going and doing”. The present study revisits the model presented by Bryant and colleagues, exploring the suitability of the model in terms of mental well-being among the oldest old (80+) in a Finnish context.

This Finnish case study set out to examine how participants aged 80 years old or over describe their mental well-being and what it entails, as perceived in their everyday lives. Based on the work by Bryant et al. (2001), the assumption was that psychosocial factors (e.g., perceived social support, close social ties and meaningful social activities) would be identified as key components of mental well-being in the narratives. The current study sought to deepen the understanding of the causal links between these components and depict in what way they were viewed to influence mental well-being in everyday life based on participants own descriptions.

## Methods

The data were collected within the scope of the European Welfare Models and Mental Wellbeing in Final Years of Life (EMMY) project (2017–2019). A participatory focus group data collection method was used with participants aged 80 years or older recruited from senior community centres, adult daycare centres, and nursing homes. These centres were carefully selected in order to obtain a broad representation of older individuals with differing levels of functioning. Detailed information on the study design, participant inclusion criteria and recruitment methods, as well as data collection procedures can be found elsewhere (Lara et al., 2019).

### Participants

The current study includes data resulting from six focus group interviews which were conducted in the Ostrobothnia region of Western Finland in the spring of 2017. Ethical approval for this study was obtained from the Ethics Research Committee at the National Institute for Health and Welfare THL, Finland (reference number THL/610/6.02.01/2017). Written informed consent was obtained from all participants (two individuals provided recorded verbal consent due to difficulties in mobility).

The interviews included 28 participants (19 women and 9 men), with a mean age of 86 years. Descriptive information on the study participants can be viewed in Table I. Eleven of the participants were recruited from senior activity centres, eight from day centres, and nine participants were recruited from nursing home contexts in the community. Eighteen participants reported living alone, while only one of the participants reported living with a spouse. Nine of the participants lived at a nursing home. With regards to health status, sixteen of the participants reported that they rated their health as good (n = 12) or very good (n = 4), while ten rated their health status as fair and two as poor. Furthermore, when asked if participants felt hampered in their daily activities, six participants said that they felt very hampered and eighteen reported that they were hampered to some extent in their daily activities, while four interviewees did not experience that they were hampered at all.

**Table I.** Description of the study participants (n = 28)

Age (years)	Gender	Education	Marital status	Self-assessed health status	Experience of being hampered in daily activities	Recruitment context
95	Female	Secondary school completed	Widowed	Good	No	Nursing home
85	Male	Secondary school completed	Married/in a relationship	Fair	Yes, to some extent	Nursing home
84	Male	Secondary school completed	Married/in a relationship	Fair	Yes, very	Nursing home
92	Female	Secondary school completed	Widowed	Good	Yes, to some extent	Nursing home
89	Female	Secondary school completed	Widowed	Fair	Yes, to some extent	Nursing home
94	Female	Secondary school completed	Widowed	Bad	Yes, very	Nursing home
87	Female	Secondary school completed	Widowed	Bad	Yes, very	Nursing home
94	Female	Secondary school completed	Divorced/separated	Fair	Yes, to some extent	Nursing home
82	Female	Secondary school completed	Widowed	Fair	Yes, to some extent	Nursing home
86	Female	Secondary school completed	Divorced/separated	Good	Yes, to some extent	Senior social/activity centre
84	Female	Secondary school completed	Widowed	Fair	Yes, to some extent	Senior social/activity centre
85	Female	Primary school completed	Widowed	Very good	No	Senior social/activity centre
84	Female	Primary school completed	Widowed	Fair	Yes, to some extent	Senior social/activity centre
88	Female	Secondary school completed	Widowed	Very good	No	Senior social/activity centre
88	Male	Secondary school completed	Widowed	Very good	Yes, very	Senior social/activity centre
84	Female	Secondary school completed	Widowed	Fair	Yes, to some extent	Senior social/activity centre
79*	Male	Primary school completed	Widowed	Good	Yes, to some extent	Senior social/activity centre
86	Male	Tertiary education completed	Married/in a relationship	Very good	Yes, to some extent	Senior social/activity centre
85	Female	Secondary school completed	Widowed	Good	No	Senior social/activity centre
82	Female	Secondary school completed	Widowed	Fair	Yes, to some extent	Senior social/activity centre
84	Male	Tertiary education completed	Widowed	Good	Yes, to some extent	Adult day care centre
85	Female	Secondary school completed	Widowed	Good	Yes, to some extent	Adult day care centre
79*	Male	Secondary school completed	Widowed	Fair	Yes, very	Adult day care centre
82	Female	Secondary school completed	Widowed	Good	Yes, to some extent	Adult day care centre
82	Male	Primary school completed	Widowed	Good	Yes, to some extent	Adult day care centre
84	Female	Primary school completed	Widowed	Good	Yes, to some extent	Adult day care centre
84	Male	Secondary school completed	Widowed	Good	Yes, very	Adult day care centre
81	Female	Secondary school completed	Widowed	Good	Yes, to some extent	Adult day care centre

*This respondent would turn 80 years old during the year the study was conducted.

### Data collection and analysis

The interviews were facilitated by three trained health science researchers, with previous experience in various interview methods. Also, the researchers have previous experience in conducting focus group interviews specifically with older participants on issues related to mental wellbeing. The interviews encompassed a structured interview protocol with open-ended questions relating to the meaning of the mental well-being concept, well-being in everyday life, as well as the participants’ views and experiences related to their peers’ mental well-being (see Lara et al., 2019 for detailed description of the interview protocol used). The study participants were encouraged to freely discuss these topics and their experiences during the group meetings, which lasted for between 55 and 85 min. The interviews were recorded, resulting in 69 pages of transcripts to be analysed.

The transcripts from the six focus group interviews were analysed using qualitative content analysis, where both manifest and latent content was considered, a procedure inspired by Graneheim, Lindgren, and Lundman (2017) and Bengtsson (2016). Initially, data extraction was performed by means of *meaning condensation* where the principal researchers read through all transcripts several times independently highlighting words, phrases and paragraphs (i.e., meaning units) considered to contain interrelated information in relation to the research question. The second step involved condensing the identified units of meaning (i.e., the number of words in meaning units were reduced while keeping the sense of the unit) and assigning them codes through an inductive process. At this point, units of meaning were defined clearly in order to enable identification of concepts and allowing material to be grouped into main categories. By the process of constant comparison, similar condensed units were assembled into four main categories. Furthermore, our synthesized data and developed categories were interpreted in relation to the model developed by Bryant et al. (2001) in order to delineate similarities and differences between the depicted dimensions from the two studies.

## Results

The participants’ views of what the concept of mental well-being encompasses were collated along the four main categories: *Activities*—enjoyment and fulfilment; *Capability*—functioning and independence; *Orientation*—awareness, shifted perspectives and values and *Connectedness*—sense of belonging. The four categories are described in detail below, accompanied with selected quotes, which seek to provide illustrative insights from the participants’ everyday experiences. Further, the descriptions offer comparisons to the model advocated by Bryant et al. (2001), as well as a synthesis of commonalities that seem to jointly support mental well-being and related experiences among the oldest old.

### Activities—enjoyment and fulfilment

In the model for healthy ageing put forward by Bryant et al. (2001), one of the four identified core elements of healthy ageing was having something worthwhile and desirable to do. Similarly to the descriptions by Bryant and colleagues, many participants in the current study highlighted a range of various activities, interests, and hobbies (both social and solitary) as being important for their mental well-being. Mental well-being was generated from a sense of meaning in terms of accomplishing something and keeping busy with various tasks, in terms of being appreciated, but also in terms of enjoyment and fulfilment. Furthermore, several participants expressed how engaging in various activities of one’s choice could, in essence, also constitute mental well-being in itself, expressed by a female participant as:

For me, well-being is [doing] what interests me.

Some of the participants specifically expressed the role of different activities, both of a social nature and other activities, as compensating for feelings of emptiness caused by losing their spouse or other losses, which was perhaps more common for these participants due to their higher age. This is exemplified by a male participant’s reply when asked to describe what a good day (a day when he experiences good mental well-being) looked like:

When I think of good days, I think about when I visit (names of a couple in the same age) […]. And also visiting other acquaintances, and especially going to see (residents at a living facility), I go there sometimes, I have acquaintances there and …

Many of the quotes in the material can be interpreted as reflecting the sociocultural backdrop of this study, i.e., the Nordic context. Specific reference to these circumstances, and how they relate to mental well-being, is noted by some of the interviewees, e.g., the following citation reflecting the agrarian work ethic with a focus on practical work and related feeling of accomplishment.

I feel well when I have something to do. Because I can’t be somewhere where I don’t have an activity. […] but I will agree with (other interview participant) that when you have completed it then you really feel well, because you were able to do it after all.

The nature of other quotes can also be seen as being context-specific, e.g., the fact that driving was highlighted by many as an important activity in relation to mental well-being. This may be connected to the fact that many of the participants had lived or were still living in a rural setting where driving is an important means of transport to leisure activities and social events, for performing everyday household tasks, but also as an enjoyable activity in itself associated with a sense of independence and autonomy. The specific issue of driving was also featured in the findings of Bryant et al. (2001), but perhaps more from a functional status perspective.

Feeling well is to be able to walk for a bit and take the car and drive somewhere and have a look around. That has to do with well-being, at least for me.

In terms of the importance of being able to engage in various activities, many participants expressed how difficulties in participating due to, for example, emerging physical limitations were detrimental to their well-being causing negative feelings and a sense of loss. However, the reactions and feelings of not being able to participate and perform various activities varied greatly, with some expressing a stance of acceptance concerning their high age and its implications for especially physical functioning. Additional reflections on the role of physical health and functioning in relation to mental well-being are presented below.

### Capability—functioning and independence

A core element of healthy ageing as identified in Bryant et al.’s model (2001) was the possession of the necessary abilities to meet perceived challenges. This is reflected in the current study in terms of several participants bringing up physical health status which was perceived to exert a significant influence on well-being by influencing participants’ ability or energy to do what they had planned or wished to accomplish, as well as influencing their sense of independence and related aspects of autonomy and functioning.

In my view, if you are physically healthy, are able to live at home and take care of yourself and place both feet on the floor in the morning and get up—that feels good to me.

Again, some of these quotes may directly reflect the socio-cultural circumstance of the participants. The commonality in the current sample of living in rural areas in private houses with all this entails may heighten the threshold for asking for help or assistance and moving into other forms of housing (whether assisted or unassisted living). Several participants mentioned how various adjustments and interventions, such as moving to housing more adapted to their changing needs could alleviate decline in physical status or support their adaptation to new circumstances—creating a sense of maintained or increased independence.

Yes, also the fact that I can manage by myself, that is perhaps also a reason for why I moved here. I don’t need to ask someone to help me all the time. I can go to the grocery store by myself, even if I go ten times if I can’t carry too much at once, I can still do it by myself. And that is something that means a lot, to notice that you can manage by yourself as long as possible.

As reflecting the individual needs and expectations and overall heterogeneity of the sample, some participants expressed a pragmatic approach regarding changes in capability and subsequent effects on everyday life. In line with this, various supportive tools related to mobility or similar were mentioned as useful, while others voiced frustrations. These inter-individual differences regarding functional abilities specifically perhaps also form a part of the orientation dimension of well-being, which in itself is a much broader element.

### Orientation—awareness, shifted perspectives and values

One of the four corner stones of healthy ageing defined by Bryant et al. (2001) was individual attitudes and the optimistic or pessimistic evaluations of one’s own health. Attitudes and outlook, perspective and values were also identified as significant elements of mental well-being in this study. The narratives in the current study focused more on change rather than static personal characteristics, with descriptions focusing on the many changes faced throughout the life course, and how these resulted in a shift in perspective and attitudes. Participants talked about the losses experienced in life, such as losing relatives and friends, retiring from a lifetime of work, changing location and thereby losing social networks and everyday routines in exchange for the unfamiliar, as well as encountering ill-health and other challenges—often in relation to being either a caregiver or care receiver. Participants describe the need for a shift in perspectives and values in order to be able to manage the new changes and challenges met in later life in order to stay positive and in this way retain well-being, as illustrated here by a female participant:

True, if you really […] every day there will come an evening, always, no matter how hard it is.

The narratives also highlight how the difficulties can bring new awareness and new insights in life. Participants describe how challenges produced an increased awareness of their own limitations and an increased understanding of what is truly important to them. Furthermore, facing difficulties also reportedly reminded them of the transience of life stating that increased awareness brings a shift of perspective, and new ways of looking at oneself, one’s surroundings, and changing personal values:

And they go sailing in the summertime. It is well deserved. I have also sailed. And enjoyed it. I can’t sit in a sail boat these days, I am not able to get in it either. They should have it as good as I’ve had it.

Elements of integration and acceptance of one’s life and various experiences also emerged, where mental well-being was depicted:

I would like to say that at the age we are all in, we all have a lot of experiences. We also have a lot of tough experiences [talks about her experience of cancer]. But I am humbly thankful that I have gotten through it and am feeling as well as I am today. But adversities also create strength and another perspective on life … you become humble. And make the most of the day you are given.

Participants demonstrate that they choose what to engage with in accordance with what is perceived as important, choosing not to engage in things that are not perceived as meaningful. Several participants highlight a growing need of solitude, enjoying one’s own company and being able to be alone, and a decreased need of engaging in social activities in order to maintain their mental well-being. This preference for solitude could also extend to close family members such as children and grandchildren.

I think that as long as you enjoy your own company, then you feel well. I think I am a hermit. I enjoy being alone.

Many of the older adults expressed the importance of freedom to choose how to live one’s life, doing what one wants when one wants to, and the possibility to form one’s daily life in a meaningful way:

I have to say, I have freedom. And I have to say that is a sense of well-being that is wonderful. I don’t feel tied down to anything and I don’t have to do anything, I can do exactly what I want to do.

One participant noted that mental well-being was at times prescribed by others, following expectations or stereotypical views, with participants feeling that others expected them to be a certain way or enjoy certain activities based primarily on their age. This was expressed in the following exchange between two interview participants:

Can we talk about the fact that when you retire you suddenly as a senior citizen are expected to start participating in activities and gatherings for seniors. I have never felt comfortable there. And I often ask myself why you are supposed to start enjoying accordion music or old songs and all that as you get older … ? Why can’t you just continue forward in time? Why do you have to go back in time and start there?”

[…]

”Exactly, you have to be able to choose. And not to accept everything that is new, without being forced to sit and sing old classics and all that they seem to think that seniors want to do.

### Connectedness—sense of belonging

Many participants describe the social dimension of life as the most important when it comes to mental well-being, with feelings of belonging and connectedness with others perceived as important mechanisms connecting mental well-being, social networks and activities in everyday life. At the same time, several participants reported that the number of their social contacts had significantly decreased with age, describing discontinued relationships and losses of a spouse, other family members and close friends:

At one point there were 12 of us going out together in the summertime, or otherwise when we have get-togethers of some sort. But now there are only three of us left.

The narratives further describe how mental well-being is linked to having long-term and continuous social relationships especially, these perceived as important for the feeling of meaning in life:

And I feel that I’ve had that privilege during my whole active life, to have a group of people that you stick with through the years. I think that is valuable. […]. You have to consciously build a social circle. For me that has been very important.

These findings line up with the model of healthy ageing advocated by Bryant et al. (2001), where social resources such as valued and close relationships to family and friends emerged as one of the four central aspects of healthy ageing. However, in the current study, the link between mental well-being and social relationships was described via feelings of being valued and needed, and that helping others and contributing to others’ well-being also enhances one’s own mental well-being:

Yes, I think that you feel very pleased when you finally get going, you have been thinking for quite a while that you should visit so and so. And when you get there and see the joy in their eyes “You’re here”, they are so happy and tears running, then it feels really good in your heart.

One day a person in the council here came wearing a necklace, and I had made that necklace! I was so happy, it suited her so well, and she said that she likes it very much, and it suited her colours. It was so nice.

Simultaneously, experienced loneliness was addressed by a few participants as a threat to mental well-being. Some participants, however, emphasized the person’s own responsibility in this matter, as exemplified by this quote by one female interviewee:

A lot of people who are alone have let themselves get lonely. They start sitting alone at home and think that life is dull and that life is no longer worth living. I think I am quite positive in my nature. I was by myself also for eight years before we started seeing each other. Some single women think it is so boring, but I have to say that I have a large social circle and I am quite social I think, so I have a lot of friends and acquaintances. And it plays a large part to have a social communion, it does a lot for your well-being.

The uncomfortable feelings connected to experienced loneliness were also addressed in the study by Bryant et al. (2001), with participants expressing how they, due to their scarce and fragmented social contacts, feared that no one would notice if they had passed away. However, the current study found participants to have a more positive outlook on their social situation, expressing the importance of being and staying socially connected to other people—seemingly not experiencing this to be too challenging to achieve in their own lives.

**Figure 1.**
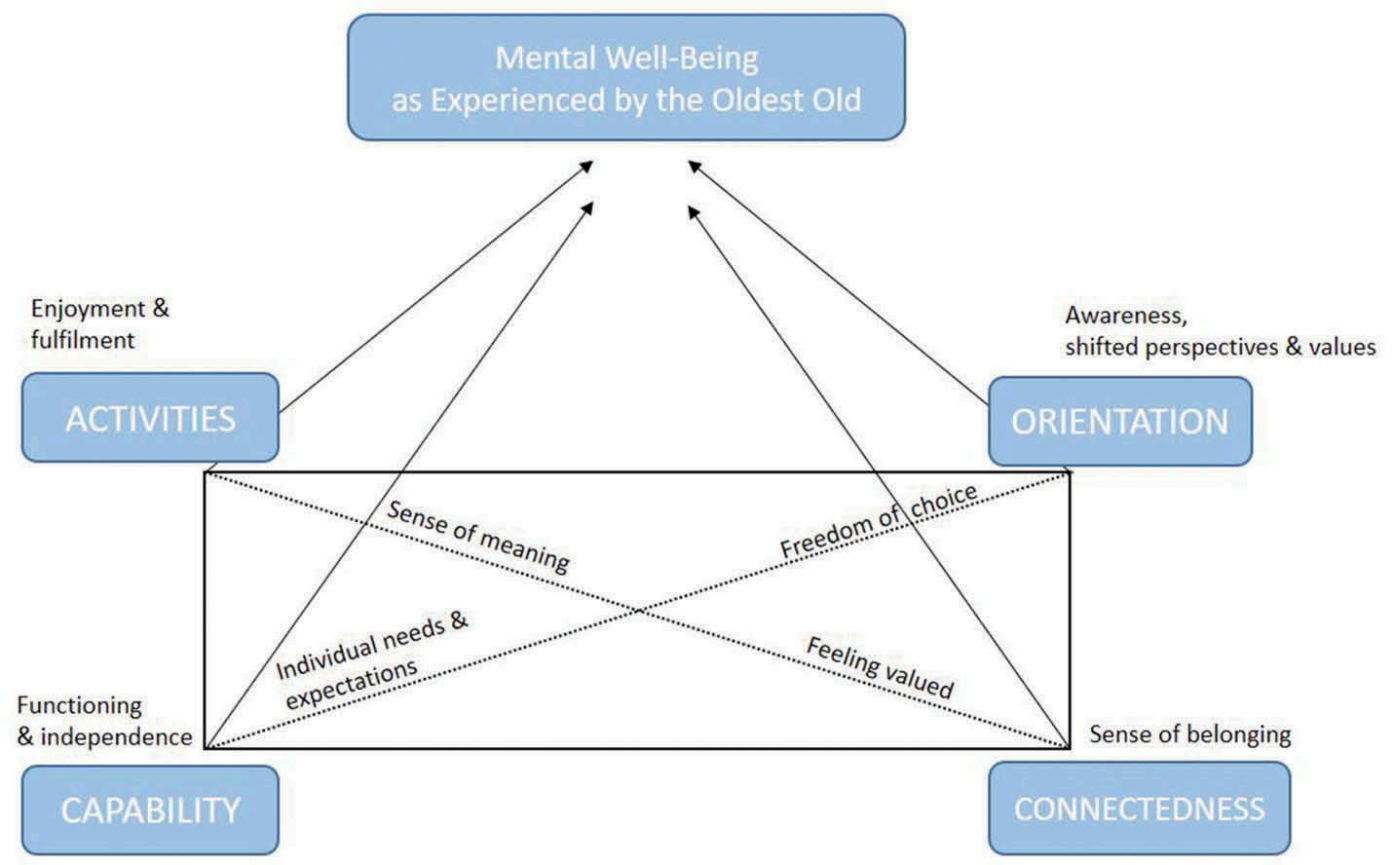
A proposed conceptualization of mental well-being among Finnish persons aged 80 years and over, adapted from Bryant et al. (2001)

Taken together, mental well-being in everyday life is perceived to encompass the four main dimensions of activities, capability, orientation and connectedness among the Finnish oldest old informants of this study. In addition to these described main categories, several commonalities could be identified from the informants’ own descriptions of what mental well-being is and means to them—despite the heterogeneous study group with regards to age, living arrangements, health and functional status and socioeconomic status alike. These were the importance of sense of meaning, freedom of choice, feeling valued, as well as accounting for individual needs and expectations (see Figure 1).

## Discussion

The current study set out to explore older adults’ perceptions with regard to what constitutes mental well-being in oldest old age. The healthy ageing model constituted a useful framework for the conceptualization of mental well-being, the current study illustrating four key components of mental well-being in oldest old age that parallel the multidimensional model of healthy ageing developed by Bryant et al. (2001); *Activities*—enjoyment and fulfilment; *Capability*—functioning and independence; *Orientation*—awareness, shifted perspectives and values; and *Connectedness*—sense of belonging. The findings highlight that although clinical health measures and related functional status undeniably play an important role for well-being in general, as often assumed in healthy ageing research and related conceptualizations—it is not the principal component of self-reported mental well-being among the oldest old. The findings highlight how many persons in this age group do not view themselves as passive or dependent, on the contrary, they underline the importance of empowering attitudes, a positive mindset and actively creating circumstances which support their mental well-being. The findings thus emphasize the subjective dimension of wellbeing, as well as the related heterogeneity between respondents in this age group transcending the age, functional status and life histories of the participants.

The socio-cultural context is assumed to influence the ageing process as well as individual experiences throughout the lifespan. Collective experiences such as rural living, agrarian work ethic emphasizing practical work, accomplishments and independence as well as historic events (e.g., war episodes in the mid-1900s) may be particularly important to mental well-being in the current context. However, as noted above, the heterogeneity of the sample may be equally, if not more, important to consider. The chronological age of the sample may carry less weight when considering their individual situation from a life-span perspective, e.g., when considering what kind of lifestyle the person had earlier on and how this has been affected by various age-related changes in factors ranging from functional level to social network. Therefore, despite the current study including a heterogeneous group of participants, they may still represent a certain demographic group in term of mental well-being. As highlighted earlier by Lara et al. (2019), it is important to consider cross-country differences in mental well-being attributed to external factors such as sociocultural phenomena and welfare systems.

The current study found autonomy, independence and experienced freedom to be particularly important, results which were also reflected in an earlier Finnish interview study (Nosraty et al., 2015), but which has not appeared as a central aspect for older adults’ mental well-being in a Southern European context (Lara et al., 2019). Supporting these elements of well-being—adhering to principles of person-centeredness (McCormack, Karlsson, Dewing, & Lerdal, 2010) with a focus on individual needs and preferences—is becoming more central not only in research and practice but also as a guiding principle within health and social care policy (the importance of which has earlier been highlighted by the World Health Organization, 2008). Finnish policy targeting older adults does today to a certain extent consider principles of inclusion, participation and individualized needs and prerequisites but this primarily in relation to social and health-care services, e.g., The Act on Supporting the Functional Capacity of the Older Population and on Social and Health Care Services for Older Persons 2012 or the Quality Recommendations to guarantee a good quality of life and improved services for older persons 2017–2019 (Ministry of Social Affairs and Health, 2018). The national Active Ageing programme 2012–2017 (The Finnish Association for the Welfare of Older People, 2017), on the other hand, which explicitly targeted promotion of mental well-being can be seen as an example of a policy considering mental wellbeing in the broad sense (as identified within this article).

Commonalities emerged between the current study and the publication by Bryant et al. (2001) which highlighted the need for including more subjective aspects such as personal attitudes and resources in conceptualizations of healthy ageing. The current study intended to develop this line of enquiry further by providing a conceptualization of mental well-being in oldest old age from a Finnish perspective, contributing to a currently unresolved understanding of the concept. Although the study by Bryant et al. (2001) provided useful basis for the current study, a few differences in approaches between the two studies are worth noting. Firstly, the current study included people of varying functional and physical health status, as well as a considerable older study sample, all aged 80 and over, while in Bryant’s study only 39.1% were aged over 75 years. The current study also used focus group methodology, while Bryant et al. (2001) used individual interviews.

### Study strengths and limitations

Focus group methodology is ideal for attaining a deeper understanding of how people think or feel about a certain theme or phenomena, although it does have its strengths and weaknesses (Krueger & Casey, 2014). Reflecting on a theme in a group setting is sometimes easier with the resulting data formed by a group process, rather than the opinions of specific individuals. However, the current topic, mental well-being, could also potentially have benefited from individual reflection and a deeper discussion which may not have been possible in a focus group interview setting. Individual interviews may provide participants with more time and space and may have yielded somewhat different results.

A particular strength of the current study was the purposeful sampling of older adults residing in different settings, meaning different levels of functioning were represented. Meaning that older people with varying level of health and related vulnerability to decreased mental well-being were included. Hence, the results may not reflect the experience of older adults in a particularly frail position including those with dementia disorders, the occurrence of which becomes more common with increasing age, a group not included in the sample. The representativeness of the sample in the current study could also be influenced by the geographical limitation of the sample (the western region of Finland) as well as the fact that the majority of the study sample was Swedish-speaking Finns. Although an official language in Finland, the Swedish-speaking language group is often identified as being more resourceful in public health studies (e.g., Nyqvist, Finnäs, Jakobsson, & Koskinen, 2008) which could limit the transferability of the findings to broader contexts, limiting the generalizability and comparability of the findings. However, this may not be too problematic considering that the intention of this study was not specifically to generalize or compare experiences of mental well-being among different older populations, but rather to theoretically elaborate on how and in what way mental well-being is conceptualized among the oldest old age group in Finland.
